# Exploring the impact, challenges, and integration of podcasts in patient education: a systematic review

**DOI:** 10.1186/s12909-025-07217-4

**Published:** 2025-05-12

**Authors:** Saeed Khayat Kakhki, Nahid Aghebati, Hossein Karimi Moonaghi

**Affiliations:** 1 Student Research Committee, School of Nursing and Midwifery, Mashhad University of Medical Sciences, Mashhad, Iran; 2 School of Nursing and Midwifery, Mashhad University of Medical Sciences, Mashhad, Iran; 3 Medical Sciences Education Research Center, Mashhad University of Medical Sciences, Mashhad, Iran; 4 Nursing and Midwifery Care Research Center, Mashhad University of Medical Sciences, Mashhad, Iran

**Keywords:** Podcast, Patient education, Health education, Media

## Abstract

**Background:**

Podcasts have become increasingly prominent as tools for health communication, offering flexible and accessible formats for patient education. While widely used in professional training, their role in supporting patient-centered learning remains underexplored.

**Methods:**

This systematic review synthesized studies published from 2010 to 2024 concerning podcast use in patient education. Five databases—PubMed, Scopus, Web of Science, Google Scholar, and Embase—were searched using defined keywords. Studies were selected based on relevance to patient education through podcasts, and outcomes such as knowledge retention, comprehension, and engagement. Data extraction was performed independently by two reviewers. Quality assessment was conducted using the Cochrane Risk of Bias Tool, the Newcastle-Ottawa Scale, and the CASP checklist. A thematic synthesis approach was employed to analyze qualitative and quantitative findings.

**Results:**

Of the twenty-one included studies, seven demonstrated improved patient knowledge retention, comprehension, and engagement through podcast use. Five studies emphasized accessibility and learner autonomy, highlighting the benefits of asynchronous and flexible delivery. Three studies explored the integration of podcasts with traditional teaching methods, showing positive outcomes when used as complementary tools. However, three studies identified challenges including content quality variability, limited production standards, and digital access disparities. Thematic synthesis categorized findings into four domains: educational effectiveness, integration with traditional education, accessibility and learner autonomy, and implementation challenges.

**Conclusions:**

Podcasts represent a promising adjunct to patient education. Their effectiveness depends on thoughtful design, contextual relevance, and equitable delivery. Standardization, blended learning models, and ongoing evaluation are essential for maximizing their impact.

**Clinical trial number:**

Not applicable.

**Supplementary Information:**

The online version contains supplementary material available at 10.1186/s12909-025-07217-4.

## Introduction

Podcasts have emerged as a transformative instrument in medical education, particularly in the context of patient education. Their capacity to convey content in an accessible and engaging manner has established them as a valuable resource for disseminating health information to a diverse audience, including patients [1, 2]. As digital tools continue to facilitate connectivity between healthcare providers and patients, podcasts present flexible, user-friendly educational content, which is especially advantageous in patient education [1]. Educational podcasts can be categorized into several formats, such as instructional, narrative, interview-based, and monologue-style episodes. Some also include visuals (video podcasts or vodcasts) that enhance comprehension through images or diagrams. This variety supports different learning preferences and literacy levels [3, 4]. This study systematically explores podcasts’ role in enhancing patient education, emphasizing their impact on knowledge retention, patient engagement, and health literacy.

Although podcasts have gained significant recognition for their influence on medical education for professionals and students, their utilization in patient education is an area of increasing interest [5–7]. Research demonstrates that podcasts have the potential to substantially enhance patients’ comprehension of intricate health subjects and facilitate the retention of essential medical information, mainly when the content is organized in a suitable manner [8–10]. Unlike traditional educational methods, like face-to-face sessions or written materials, podcasts allow patients to access educational content whenever they want, making learning more flexible to fit their schedules and needs [4, 11].

While podcasts have demonstrated substantial potential in enhancing medical education for professionals, their influence on patient education has garnered comparatively less attention. Several studies published in BMC Medical Education have also highlighted the utility of podcasts in educational contexts, particularly among students and healthcare professionals (e.g., Prakash et al., 2017; Schreiber et al., 2010) [12, 13]. These studies reinforce the need to further examine podcast-based learning in the patient education context.

Despite these advantages, incorporating podcasts into patient education poses several challenges. Primary concerns include inconsistencies in content quality, the absence of standardized production guidelines, and limited access to technology and internet infrastructure, particularly in resource-limited settings [14, 15]. These barriers can hinder the effectiveness and reach of podcast-based education. However, this review also identifies significant opportunities to enhance the quality and accessibility of educational podcasts, such as developing frameworks for evaluating content quality and creating evidence-based resources to ensure reliability [16, 17].

In patient education, podcasts have proven to be an effective means of disseminating health information to both healthcare providers and patients. For instance, initiatives like Radio-Salmandan for older adults and PediaCast CME have successfully utilized podcasts to communicate pediatric content to healthcare professionals, enhancing their knowledge and improving patient care [16, 18]. The ability to connect with a broad audience through digital platforms enhances the effectiveness of podcasts in advancing health literacy for patients and caregivers [17].

Although a number of systematic and scoping reviews have investigated the role of podcasts in medical and professional education, their application in patient education remains largely underexplored. For instance, Cho et al. (2017) reviewed the use of podcasts in undergraduate medical curricula, emphasizing feasibility but providing limited discussion on patient-facing outcomes [9]. Kelly et al. (2022) conducted a scoping review on podcast use in clinical education, focusing on knowledge translation for students and practitioners [4]. Similarly, Caldwell et al. (2024) synthesized findings across medical education contexts but did not include patient engagement or comprehension metrics [19]. Collectively, these reviews underscore the growing relevance of podcasts in education but do not systematically address their role in enhancing patient learning, health literacy, or engagement. This gap provides a compelling rationale for the present review, which focuses explicitly on podcasts in patient education.

To address this gap, the current review explores the impact, challenges, and integration of podcasts in patient education by answering the following questions.

1. How do podcasts impact knowledge retention, engagement, and comprehension in patient education?
2. What challenges affect the use of podcasts in delivering patient education?
3. How are podcasts integrated into broader educational strategies in healthcare?

## Methods

This study is a systematic review assessing podcasts’ impact, challenges, and integration in patient education. It adheres to established systematic review protocols and guidelines, particularly the PRISMA (Preferred Reporting Items for Systematic Reviews and Meta-Analyses) framework [20]. This review synthesizes literature published between 2010 and 2024 to assess how podcasts contribute to patient education, with a focus on their effects on knowledge retention, patient engagement, accessibility, and integration into traditional education frameworks. The timeframe (2010–2024) was chosen to reflect the emergence and rapid expansion of podcasting as an educational tool in healthcare. Literature published prior to 2010 is either nonexistent or too sparse and anecdotal to be systematically evaluated. Several reviews and empirical studies on podcasting in medical education began appearing around 2010, marking a significant shift in its adoption and integration into educational strategies.

This systematic review was not pre-registered in PROSPERO or other public registries. While protocol registration is an important element in ensuring transparency and reducing reporting bias, we opted not to register due to the exploratory nature of the review and the iterative development of the inclusion criteria and thematic categories during early screening stages. Nonetheless, to enhance methodological transparency, we adhered closely to the PRISMA 2020 guidelines and provided detailed documentation of our eligibility criteria, data extraction methods, risk of bias assessment, and synthesis strategy.

### Information sources

To identify pertinent studies, a systematic search of five principal electronic databases was performed. The databases utilized in this search include PubMed, Scopus, Web of Science, Google Scholar, and Embase, all esteemed for their extensive coverage of health, medicine, education, and social sciences.

### Search strategy

A comprehensive search strategy was developed for each database using relevant keywords and Boolean operators. This strategy aimed to capture articles discussing the role of podcasts in patient education and their effects on health literacy and learning outcomes. Detailed search strategies for each database are provided in the supplementary file.

### Eligibility criteria

Studies were included if they met the following criteria:

1. **Focus**: Articles that podcasts are directly examined for educating patients, improving health literacy, or enhancing patient engagement. Studies primarily related to professional medical education were also included if they presented transferable findings relevant to patient learning (e.g., flexibility, accessibility, comprehension).
2. **Study Design**: Quantitative and qualitative studies—including experimental, observational, systematic reviews, and case studies—were eligible.
3. **Outcomes**: Studies reporting on at least one of the following: knowledge retention, comprehension, patient engagement, satisfaction with podcast-based learning, or behavioral changes resulting from podcast exposure.
4. **Timeframe**: Only studies published between 2010 and 2024.
5. **Language**: English only.

Studies were excluded if they:

(1) focused solely on professional education with no patient-related insights. One included study—Caldwell et al. (2024)—was a previously published systematic review. It was retained not for data extraction or coding, but solely to contextualize an existing theme in the Results section. No findings from this review were included in the thematic synthesis;

(2) lacked empirical data or a defined methodology (e.g., commentaries, letters to the editor, editorials, and other non-empirical publications were excluded unless they presented empirical data or structured methodology);

(3) were not in English;

or (4) lacked sufficient outcome information to assess relevance.

### Data extraction and management

Data extraction was conducted independently by two reviewers using a standardized form. The extracted data included:

- Study Characteristics– author(s), year of publication, study design, sample size, and setting.
- Podcast Characteristics– type and format (e.g., informational, instructional, or narrative), duration, frequency, and topics covered (e.g., chronic disease management, medication adherence).
- Outcomes– knowledge retention, engagement, comprehension, and patient satisfaction.
- Methodological Details– tools used to assess patient outcomes (e.g., surveys, interviews, knowledge tests) and analytical approaches.

Initial reference records were organized using Google Sheets and later managed with EndNote, for deduplication and citation control. The search strategy was developed by the lead author and refined in consultation with two senior team members with expertise in literature synthesis. Although no formal librarian was involved, the final strategy was peer-reviewed internally.

Any disagreements in study selection or data extraction were resolved through discussion or third-party adjudication. These procedures ensured consistency, transparency, and methodological rigor throughout the review process.

### Quality assessment

Given the methodological diversity of the included studies, we used different validated tools based on study design to ensure appropriate and rigorous assessment: For randomized controlled trials (RCTs), we applied the Cochrane Risk of Bias 2 (RoB 2) tool. For observational studies, the Newcastle-Ottawa Scale (NOS) was used. And for qualitative studies, we applied for the Critical Appraisal Skills Programme (CASP) checklist. This stratified approach allowed us to assess each study with the most appropriate and design-specific tool, enhancing the precision and relevance of our quality assessment. All reviewers followed standardized scoring rubrics and discussed discrepancies collaboratively to ensure consistency. Studies were not excluded based on their risk of bias ratings. However, the assessments informed the narrative synthesis, and findings from studies rated as moderate or high risk were interpreted with appropriate caution during analysis. More information is provided in Table S1 (Supplementary File).

### Data synthesis

Due to heterogeneity in study designs and outcome measures, we employed a thematic synthesis approach to qualitatively analyze and interpret findings across studies. The thematic analysis followed the six-phase framework outlined by Braun and Clarke (2006): [1] familiarization with the data [2], generating initial codes [3], searching for themes [4], reviewing themes [5], defining and naming themes, and [6] producing the report [21].

All included studies were read and coded independently by two reviewers using an inductive approach. Themes were derived based on recurring patterns in the data. Any discrepancies were resolved through discussion or with the help of a third reviewer. This method allowed us to identify and synthesize key concepts across the studies into coherent thematic categories.

The resulting themes included topics such as accessibility, flexibility, knowledge retention, patient engagement, and the complementary role of traditional teaching methodologies. Additionally, challenges such as variability in content quality, limited access to technology, and demographic disparities were also explored. The results were presented narratively, emphasizing both the benefits and limitations of podcast integration into patient education.

### Limitations

Numerous limitations have been identified within the studies included in this review: (1) Heterogeneity in Study Designs: The variations observed in research methodologies, such as discrepancies in sample sizes, outcome measures, and podcast formats, have impeded the ability to undertake a comprehensive meta-analysis. (2) Quality of Evidence: Several studies were assessed to be at moderate or high risk of bias, potentially compromising their findings’ robustness. (3) Geographic and Technological Barriers: Restricted access to essential technology and internet infrastructure in certain regions may limit the generalizability of podcast-based educational initiatives. (4) Relevance of Some Included Studies: A few studies were conducted in professional education contexts. However, they were retained in this review because they offered transferable insights applicable to patient education—such as strategies for enhancing comprehension, engagement, and content delivery. This decision is acknowledged as a methodological limitation but also reflects the scarcity of empirical studies focusing exclusively on patient education through podcasts. (5) Lack of Protocol Registration: This review was not pre-registered in PROSPERO. Although protocol registration improves transparency, the exploratory scope of the study and evolving inclusion criteria led us to forego pre-registration. We acknowledge this as a limitation and have followed PRISMA standards to ensure rigor in reporting.

## Results

**Fig. 1:**
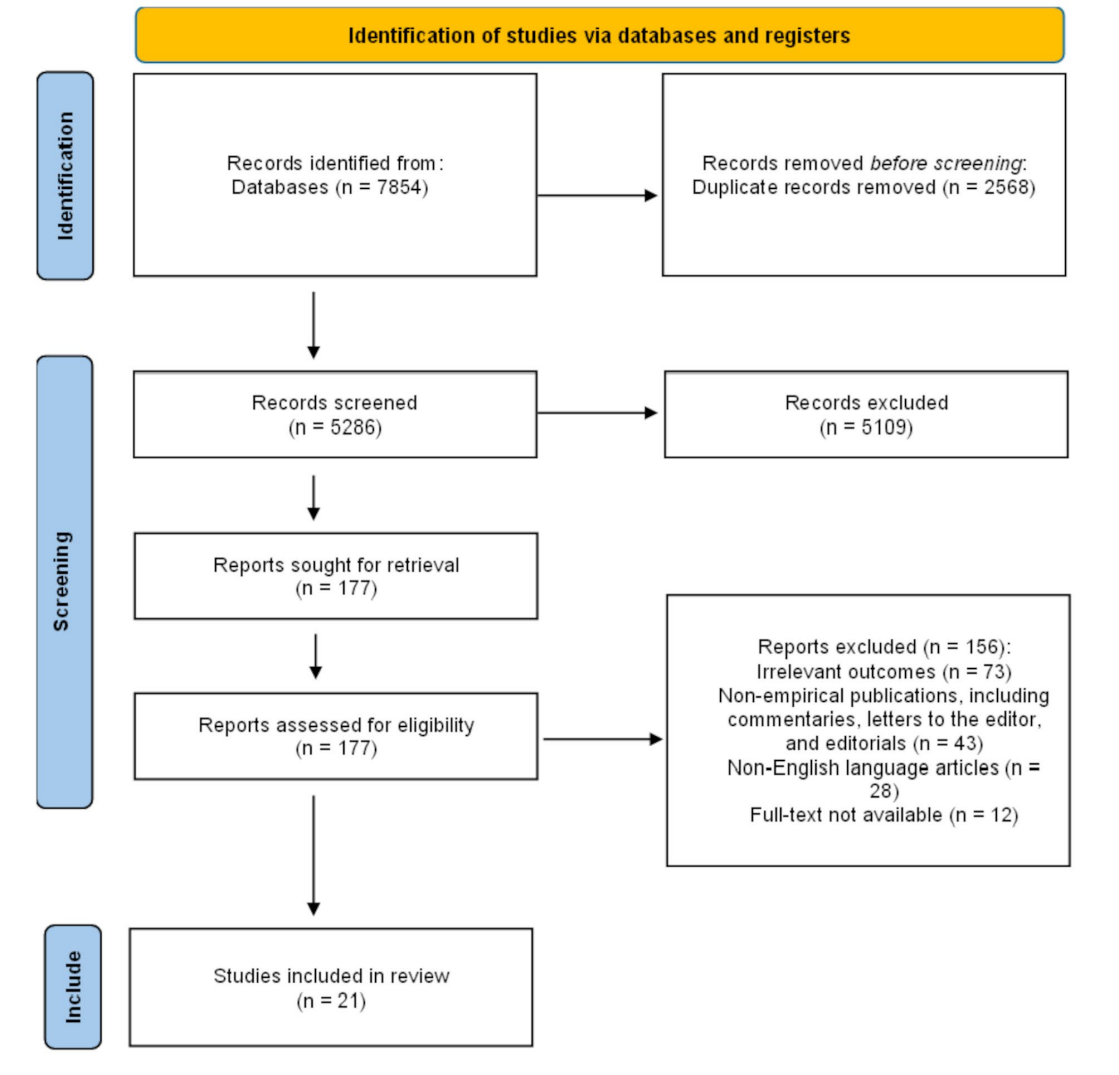
PRISMA flow diagram

A total of 21 articles were ultimately included in the review after a thorough screening process. Initially, a broad search across five key databases identified numerous studies on podcast use in patient education. A detailed flowchart following the PRISMA guidelines, illustrating the study selection process, is provided below in Fig. 1. The flowchart outlines the number of records identified, screened, eligible, and ultimately included in the review, along with the reasons for excluding certain studies at each stage. More information about the characteristics of included studies is provided in Table 1.

Incorporating podcasts into patient education has shown significant potential for improving learning outcomes, offering benefits across several important aspects of patient education. The reviewed literature provides key insights into the effectiveness and challenges of using podcasts for patient education:

### Effectiveness of podcasts in patient education

Among the 21 included studies, 7 studies reported positive educational outcomes from podcast use, particularly in enhancing knowledge retention, comprehension, and learner engagement. Podcasts were widely regarded as a flexible and accessible modality that supports patient learning at an individualized pace. For instance, Kelly et al. (2022) emphasized the value of podcasts for improving knowledge and behavior, noting their portability and efficiency [4]. Similarly, Bensalem-Owen et al. (2011) found that podcast-based instruction was as effective as traditional lectures in conveying technical EEG content [22]. Michael W. (2020) further demonstrated improved knowledge retention when interpolated questions were embedded within podcasts, highlighting their potential for active learning [15]. In the context of nursing education, Mitchell et al. (2021) observed significant gains in student confidence and knowledge following exposure to a podcast on delirium [23]. These findings collectively suggest that when structured effectively, podcasts can substantially reinforce learning, particularly for complex or technical health topics.

### Challenges and limitations in podcast implementation

Despite their promise, 3 studies highlighted substantial challenges in using podcasts as an educational tool. The most frequently cited issue was the lack of content standardization and quality control, which may reduce credibility and educational value. For example, Kane et al. (2019) criticized the inconsistent quality across drug-related podcasts, noting the underrepresentation of pharmacist perspectives and the absence of peer review mechanisms [24]. Caldwell et al. (2024) echoed these concerns, stating that while podcasts are widely used, robust evidence supporting their efficacy remains limited [19]. Technological barriers also emerged as a theme, especially in studies that addressed digital access disparities. Kevin John M. (2021) noted that while student-run podcasts promote inclusivity and mentorship, not all learners have equal access to the necessary devices or platforms [10]. These findings point to the need for guidelines, quality benchmarks, and infrastructure support to ensure equitable podcast-based education. Some studies also highlighted socioeconomic and geographic disparities in access, suggesting the need for more inclusive podcast delivery strategies in low-resource settings.

### Integration with traditional educational strategies

A smaller subset of studies (3 out of 21) explored how podcasts could be integrated with traditional teaching methods. These studies viewed podcasts not as replacements, but rather as complementary tools that extend and reinforce core instructional content. Schreiber et al. (2010) conducted a randomized trial comparing podcasts to live lectures and found no significant differences in knowledge recall, although students preferred the interactivity of live sessions [13]. Roth et al. (2020) similarly concluded that both audio and written formats were equally effective in knowledge transfer, but podcasts were rated as more enjoyable and flexible [25]. Moreover, Rachel M. (2022) and Kevin John M. (2021) highlighted the role of podcasts in fostering professional identity, wellness, and peer mentoring—features especially valuable in community-based or self-directed learning environments [5, 10].

### Accessibility and convenience

A recurring advantage reported across studies was the high accessibility and flexibility that podcasts offer. Patients and learners could engage with content at their own pace and in diverse settings, whether commuting, at home, or during breaks. This asynchronous format is especially helpful for individuals with limited time, mobility constraints, or those unable to attend in-person education. For example, Mitchell et al. (2021) and Roth et al. (2020) emphasized that the ability to pause, replay, and revisit episodes contributed significantly to knowledge retention and comprehension [23, 25]. Rachel M. (2022) further noted that learners appreciated the convenience and autonomy provided by podcasts, fostering a sense of ownership in their educational journey [5]. These aspects are particularly relevant for patient education, where personalized pacing and availability are crucial for understanding complex medical information.

### Summary of educational themes

Thematic synthesis revealed several recurring patterns regarding podcast use in patient education. Based on our analysis of the included studies, the following thematic categories were identified:

- Educational effectiveness (7 studies): Several studies highlighted improved knowledge retention, comprehension, or engagement following exposure to well-structured podcasts [4, 11, 13, 15, 22, 23, 25].
- Complementary role with traditional education (3 studies): A few studies emphasized podcasts as supplementary tools that reinforce or extend face-to-face or written instruction [25–27].
- Accessibility and learner autonomy (5 studies): Podcasts were praised for supporting self-paced, asynchronous learning and overcoming time or location constraints [5, 10, 11, 27, 28].
- Implementation challenges and quality concerns (3 studies): Some studies identified issues related to unequal access to technology, lack of content standardization, and variability in production quality [10, 19, 24].

#### Note

Several studies contributed to more than one thematic category; numbers reflect the primary thematic alignment as classified in Table 1.

These findings collectively support the integration of podcasts into patient education strategies, particularly when paired with clear guidelines and high-quality production practices. A detailed summary of included studies, along with their thematic classification, is provided in Table 1.

**Table 1:** Characteristics and thematic classification of included studies this table combines the core characteristics of the 21 included studies with their thematic classification, as identified during the synthesis. Themes reflect the studies’ primary educational focus: effectiveness, challenges, or integration

No.	Title	First Author	Publication Year	Journal	Study Design	Sample Size	Outcome	Theme
1	An Evaluation of Emergency Medicine Core Content Covered by Podcasts	Riddell J.	2023	Western Journal of Emergency Medicine	Retrospective review	N/A	Imbalanced coverage of EM core topics; gaps in musculoskeletal, hematology, and environmental content.	Other
2	Brain development, mental health and addiction: a podcast series for undergraduate medical education	J. MacDonald C.	2013	Interactive Technology and Smart Education	Qualitative Research	19	Positive reception for podcast use in medical education; suggestions for improvement implemented to meet user needs.	Other
3	Podcasting in medical education: a review of the literature. Korean journal of medical education	Cho D.	2017	Korean journal of medical education	Review of the literature	N/A	Podcasts are feasible and accepted by learners; limited evidence on efficacy and best practices; need for rigorous studies on behavior and patient outcomes.	Other
4	Learning through listening: a scoping review of podcast use in medical education	Kelly JM.	2022	Academic Medicine	Scoping review	N/A	Podcasts valued for portability, efficiency; improve knowledge and behavior; no data on patient outcomes.	Effectiveness
5	Health care professional and caregiver attitudes toward and usage of medical podcasting: questionnaire study	Lee C.	2022	JMIR pediatrics and parenting	Survey study	251	Health professionals engage more than parents; both value accuracy, transparency, and credibility.	Other
6	Short-duration podcasts as a supplementary learning tool: perceptions of medical students and impact on assessment performance	Prakash S.	2017	BMC medical education	Pre- and post-intervention	94	Short podcasts well-received; useful for revision; no overall score difference.	Other
7	Podcasts as an integral part of free open access medical education	Fernandes CAdS.	2023	Revista Brasileira de Educação Médica	Narrative review	N/A	Podcasts are a promising complementary tool; need evidence-based guidelines for development.	Integration
8	Depth of Anesthesia: A Podcast Project to Improve Perioperative Patient Care	Hao D.	2021	Transl Perioper & Pain Med	Descriptive analysis	N/A	Podcasts promote evidence-based practice, critical thinking, and global engagement in anesthesia.	Other
9	Education research: evaluating the use of podcasting for residents during EEG instruction: a pilot study.	Bensalem-Owen M.	2011	Neurology	Pre- and post-intervention	10	Podcast training as effective as traditional lectures for EEG knowledge improvement.	Effectiveness
10	Evaluation of a delirium awareness podcast for undergraduate nursing students in Northern Ireland: a pre−/post-test study	Mitchell G.	2021	BMC nursing	Pre- and post-intervention study with questionnaires assessing knowledge and confidence.	320	Podcast improved nursing students’ delirium knowledge and confidence significantly.	Effectiveness
11	Why not a podcast? Assessing narrative audio and written curricula in obstetrical neurology	Roth J.	2020	Journal of Graduate Medical Education	Randomized controlled trial	60	Podcasts and written materials improved knowledge equally; podcasts rated more enjoyable.	Effectiveness
12	Student-Led Medical Education Podcast Improves Academic Preparedness, Increases Sense of Belonging, and Enhances Wellness	Rachel M.	2022	Research Square	Survey	143	Student-run podcasts reduce stress, increase preparedness, and foster belonging.	Other
13	Characteristics of drug-related podcasts and this medium’s potential as a pharmacy education tool	Kane SP.	2019	American Journal of Pharmaceutical Education	Descriptive analysis	N/A	Drug-related podcasts are accessible but lack quality control; pharmacists underrepresented.	Challenges
14	How to create and evaluate a resident-led audio program: six clinical podcasts for medicine house staff	Ghiathi C.	2020	MedEdPORTAL	Pre- and Post-Intervention Study	184	Podcasting can be a resource for resident education and an opportunity for residents to grow as medical educators.	Other
15	Listen up: a systematic review of the utilization and efficacy of podcasts for medical education	Caldwell KE.	2024	Global Surgical Education-Journal of the Association for Surgical Education	Systematic review	N/A	Podcasts widely used but efficacy unclear; limited high-quality evidence supports their use.	Challenges
16	Live lecture versus video podcast in undergraduate medical education: A randomised controlled trial	Schreiber BE.	2010	BMC medical education	Crossover randomized controlled trial	100	No difference in knowledge recall; students preferred live lectures over podcasts.	Effectiveness
17	Creation of a Student-Run Medical Education Podcast: Tutorial	Kevin John M.	2021	JMIR medical education	Descriptive study	N/A	Student-run podcasts foster professional identity and near-peer mentoring; widely accessible.	Challenges
18	Effect of Interpolated Questions on Podcast Knowledge Acquisition and Retention: A Double-Blind, Multicenter, Randomized Controlled Trial	Michael W.	2020	Annals of Emergency Medicine	Double-blind randomized controlled trial	137	Interpolated questions in podcasts improve knowledge retention, especially for highlighted material.	Effectiveness
19	Educational Impact of a Podcast Covering Vitreoretinal Topics: 1-Year Survey Results	Michael J.	2019	Journal of VitreoRetinal Diseases	Cross-sectional survey	16,016	Podcasts valued for staying updated and learning; no replacement for traditional methods.	Effectiveness
20	Texting brief podcasts to deliver faculty development to community-based preceptors in longitudinal integrated clerkships	Bernstein J.	2018	MedEdPORTAL	Pre- and post-survey	33	Podcasts improved teaching practices; well-received by community-based preceptors.	Integration
21	A new podcast for reducing stigma against people living with complex mental health issues: Co-design study	Carrotte E.	2023	Journal of Survey in Fisheries Sciences	Mixed methods study (Cross-Sectional Survey & Qualitative Focus Groups)	25	reduce stigma through lived experience narratives.	Other

## Discussion

This systematic review offers a nuanced synthesis of the impact, challenges, and integration of podcasts in patient education, using findings from 21 studies spanning diverse methodologies and contexts. Our analysis provides timely insights into how podcast-based interventions can support patient learning and engagement, while also revealing the conditions under which their effectiveness is optimized or limited.

### Effectiveness of Podcast-Based education

A core finding of this review is that 7 out of 21 studies demonstrated positive educational outcomes associated with podcast use, especially regarding comprehension, knowledge retention, and learner engagement. For instance, Bensalem-Owen et al. [22] found podcast instruction as effective as live EEG lectures, while Weinstock et al. [15] reported that interpolated questioning within podcasts significantly improved knowledge acquisition. These results align with Mitchell et al. [23], whose study highlighted notable improvements in nursing students’ knowledge following exposure to a delirium-focused podcast.

Importantly, effectiveness was strongly linked to podcast design quality. As emphasized by Roth et al. [25], narrative structure and content relevance shaped user engagement, while Kelly et al. [4] stressed the value of concise, portable formats for patient and caregiver learning. Hao et al. [26] further illustrated how podcast initiatives in perioperative care fostered critical thinking and global knowledge dissemination, supporting their role in enhancing reflective, evidence-informed learning environments—even in clinical contexts.

These findings suggest that the medium itself does not guarantee educational success—rather, its instructional value emerges when aligned with intentional design strategies and evidence-based content.

### Accessibility and learner autonomy

Five studies in this review emphasized the role of podcasts in supporting learner autonomy and enhancing accessibility. For instance, Rachel et al. [5] and Kevin John et al. [10] reported that podcasts enabled users to learn at their own pace, accommodating varying schedules and responsibilities. Venincasa et al. [11] highlighted the benefits of asynchronous access for reinforcing understanding, particularly in contexts involving complex or evolving health information. Bernstein et al. [27] demonstrated the practical advantage of delivering concise podcast episodes via mobile messaging, improving engagement in remote learning environments. Ghiathi et al. [28] also described how podcast creation and participation empowered resident learners, supporting autonomy and near-peer collaboration.

These findings suggest that podcasts, through flexible and self-paced delivery, can offer meaningful support for patient education—particularly when traditional formats are inaccessible or overwhelming.

### Challenges in implementation and access

Despite their promise, three studies identified substantial limitations in the practical use of podcasts for educational purposes. Kane et al. [24], for example, documented significant inconsistencies in podcast content quality, while Caldwell et al. [19] noted the overall lack of standardized development practices across the field. Kevin John et al. [10] further reported disparities in access among learners, particularly those from underserved communities lacking reliable internet or personal devices—an issue that Carrotte et al. [2] linked to broader patterns of digital health inequity. In addition, Fernandes et al. [29] emphasized the absence of clear guidelines for podcast development, noting that this gap hampers the reliability and pedagogical consistency of podcast-based education. Their narrative review highlighted the need for evidence-based frameworks to guide podcast design in both professional and patient education settings.

Additionally, podcasts inherently lack interactive elements such as real-time feedback or adaptive clarification, which are often critical for patient understanding, especially in cases involving complex medical decisions or emotional sensitivity. Unlike face-to-face education or even synchronous online formats, podcast listeners cannot easily engage in two-way communication to resolve uncertainties. This one-directional nature, while supporting flexibility, may also limit deeper comprehension or discourage active learning in some patient populations [25].

Such findings reinforce the notion that access and equity are not guaranteed by the digital nature of podcasts. Without deliberate infrastructure planning and inclusive design, podcast initiatives risk amplifying, rather than reducing, educational gaps.

### Integration with traditional modalities

Three studies in the review examined podcasts as complementary to, rather than replacements for, traditional educational strategies. Schreiber et al. [13] demonstrated comparable learning outcomes between podcast and live lecture groups, though students preferred the interactive nature of in-person sessions. Similarly, Bernstein et al. [27] found that brief podcast episodes texted to community-based preceptors could reinforce key content while preserving time flexibility.

From a theoretical standpoint, podcasting resonates with constructivist learning paradigms, promoting self-paced, reflective engagement (as noted by Mitchell et al. [23]). However, as Ghiathi et al. [28] observed, podcasts may lack the immediacy of feedback and dialogical engagement central to live instruction. Therefore, a blended model appears optimal—using podcasts to augment, not substitute, real-time education.

### Thematic and contextual implications

Thematically, this review underscores the importance of accessibility, personalization, and asynchronous delivery as key strengths of podcast-based learning, particularly for patient populations facing logistical or cognitive barriers. As highlighted in studies by Rachel et al. [5] and Venincasa et al. [11], podcasts enabled learners to control their pace and revisit content as needed, a feature especially valued in chronic disease management and emotionally sensitive topics.

However, the findings also point to the need for contextual tailoring. Elderly listeners, low-literacy groups, or patients with cognitive challenges may benefit more from video-enhanced or guided listening formats, as emphasized by Alayed et al. [1] and Newman et al. [30].

### Methodological considerations

While the review followed rigorous methods, several limitations must be acknowledged. First, heterogeneity across study designs and outcomes limited the feasibility of meta-analysis. Second, nearly half of the included studies had moderate to high risk of bias, which we addressed by interpreting their findings cautiously within the synthesis. Third, although the review focused on patient education, several included studies originated in professional learning contexts; these were retained only when their findings were transferable to patient education (e.g., strategies for improving engagement, comprehension, and delivery flexibility).

Finally, the review was not pre-registered in PROSPERO or another public registry. While we followed a structured, transparent approach aligned with PRISMA guidelines, including independent dual screening and thematic synthesis, this omission is acknowledged as a limitation and should be considered in evaluating the reproducibility of our methods.

### Implications for practice and future research

The findings of this review support the inclusion of podcasts as a scalable and patient-centered educational tool, particularly when thoughtfully integrated into broader educational frameworks. Given the diversity of patients’ learning needs, podcast-based strategies should not be implemented as one-size-fits-all solutions. Instead, educators and health systems should prioritize content standardization, audience-tailored delivery, and continuous quality monitoring.

Emerging evaluation tools such as METRIQ-5 and METRIQ-8 can guide content creators in producing reliable and high-quality educational podcasts [9]. In parallel, engaging patients in co-design processes—as suggested by participatory studies like Alayed et al. [1]—can improve content relevance and accessibility, particularly for underserved groups.

Future research directions should include:

- Rigorous testing of behavioral and long-term clinical outcomes associated with podcast use.
- Comparative effectiveness studies contrasting podcasts with video, printed, or interactive learning modules.
- Exploration of blended educational models, combining podcasts with in-person or telehealth-based guidance.
- Assessment of podcast impact in non-Western, multilingual, and low-resource environments, where implementation challenges may differ significantly.

## Conclusion

This systematic review highlights the potential of podcasts as flexible, scalable tools for patient education. When designed intentionally and aligned with evidence-based principles, podcasts can enhance knowledge retention, engagement, and comprehension across diverse patient groups. However, their effectiveness is not inherent to the format and depends on content quality, contextual relevance, and equitable access. Challenges, including variability in production standards and digital infrastructure disparities—must be addressed through standardization frameworks and inclusive design. A blended educational approach, integrating podcasts with traditional modalities, appears most effective. Future initiatives should focus on rigorous evaluation, patient co-design, and broader implementation strategies to ensure that podcasting contributes meaningfully to equitable, high-quality health education.

## Electronic supplementary material

Below is the link to the electronic supplementary material.

Supplementary Material 1

Supplementary Material 2

Supplementary Material 3

## Data Availability

All data analyzed in this study were extracted from previously published studies, which are cited in the manuscript. No new datasets were generated. Therefore, data sharing is not applicable.

## Abbreviations

PRISMA: Preferred Reporting Items for Systematic Reviews and Meta-Analyses
NOS: The Newcastle-Ottawa Scale
CASP: Critical Appraisal Skills Programme
