# Supplementary material for: Exploring the impact, challenges, and integration of podcasts in patient education: a systematic review
Source: BMC Med Educ. 2025 May 12;25:690. doi: 10.1186/s12909-025-07217-4 (PMC12067963; doi:10.1186/s12909-025-07217-4)
Supplement: Supplementary file 1 — Supplementary Material 1 [file 12909_2025_7217_MOESM1_ESM.docx]

**Supplementary File**

**Search Strategy**

| **Database Name** | **Search Strategy** |
| --- | --- |
| PubMed | (((((((((((((((((((((("Patient Education"[Title/Abstract]) OR ("Health Education"[Title/Abstract])) OR ("Health Literacy"[Title/Abstract])) OR ("Patient Engagement"[Title/Abstract])) AND ("Podcasting"[Title/Abstract])) OR ("Digital Media"[Title/Abstract])) OR ("Audio Education"[Title/Abstract])) OR ("Audio Based Education"[Title/Abstract])) OR ("Health Podcasts"[Title/Abstract])) OR ("Podcast Based Education"[Title/Abstract])) AND ("Knowledge Retention"[Title/Abstract])) OR ("Comprehension"[Title/Abstract])) OR ("Memory"[Title/Abstract])) OR ("Learning Outcomes"[Title/Abstract])) OR ("Understanding"[Title/Abstract])) AND ("Digital Health"[Title/Abstract])) OR ("mHealth"[Title/Abstract])) OR ("eHealth"[Title/Abstract])) OR ("Mobile Health"[Title/Abstract])) OR ("Digital Health Literacy"[Title/Abstract])) AND ("Medical Education"[Title/Abstract])) OR ("Clinical Education"[Title/Abstract])) OR ("Professional Training"[Title/Abstract]) AND (2010:2024[pdat]) |
| Scopus | TITLE-ABS-KEY ("patient education" OR "health education" OR "health literacy" OR "patient engagement") AND TITLE-ABS-KEY ("podcasts" OR "digital media" OR "audio education" OR "audio-based education" OR "health podcasts" OR "podcast-based education") AND TITLE-ABS-KEY ("knowledge retention" OR "comprehension" OR "memory" OR "learning outcomes" OR "understanding") AND TITLE-ABS-KEY ("digital tools" OR "eHealth" OR "mHealth" OR "mobile health" OR "digital health literacy") AND TITLE-ABS-KEY ("medical education" OR "clinical education" OR "professional training") AND PUBYEAR > 2009 AND PUBYEAR < 2025 |
| Web of Science | TS=("patient education" OR "health education" OR "health literacy" OR "patient engagement") AND TS=("podcasts" OR "digital media" OR "audio education" OR "audio-based education" OR "health podcasts" OR "podcast-based education") AND TS=("knowledge retention" OR "comprehension" OR "memory" OR "learning outcomes" OR "understanding") AND TS=("digital tools" OR "eHealth" OR "mHealth" OR "mobile health" OR "digital health literacy") AND TS=("medical education" OR "clinical education" OR "professional training") AND PY=(2010-2024) |
| Google Scholar | "patient education" OR "health education" OR "health literacy" OR "patient engagement" AND "podcasts" OR "digital media" OR "audio education" OR "audio-based education" OR "health podcasts" OR "podcast-based education" AND "knowledge retention" OR "comprehension" OR "memory" OR "learning outcomes" OR "understanding" AND "digital tools" OR "eHealth" OR "mHealth" OR "mobile health" OR "digital health literacy" AND "medical education" OR "clinical education" OR "professional training" |
| Embase | ('patient education' OR 'health education' OR 'health literacy' OR 'patient engagement') AND ('podcasts' OR 'audio education' OR 'digital media' OR 'audio-based education' OR 'health podcasts' OR 'podcast-based education') AND ('knowledge retention' OR 'comprehension' OR 'memory' OR 'learning outcomes' OR 'understanding') AND ('digital health' OR 'mHealth' OR 'eHealth' OR 'mobile health' OR 'digital health literacy') AND ('medical education' OR 'clinical education' OR 'professional training') AND [2010-2024]/py |
