# Supplementary material for: Exploring the impact, challenges, and integration of podcasts in patient education: a systematic review
Source: BMC Med Educ. 2025 May 12;25:690. doi: 10.1186/s12909-025-07217-4 (PMC12067963; doi:10.1186/s12909-025-07217-4)
Supplement: Supplementary file 2 — Supplementary Material 2 [file 12909_2025_7217_MOESM2_ESM.docx]

**Supplementary File**

**Risk of Bias Assessment**

**Table 1- Risk of Bias Assessment table of included Studies**

| **No.** | **Title** | **First Author** | **Year** | **Study Design** | **Risk of Bias Tool** | **Bias Level** | **Notes** |
| --- | --- | --- | --- | --- | --- | --- | --- |
| 1 | An Evaluation of Emergency Medicine Core Content Covered by Podcasts | Riddell J. | 2023 | Retrospective review | NOS | Moderate | Potential selection bias due to retrospective nature |
| 2 | Brain development, mental health and addiction: a podcast series for undergraduate medical education | J. MacDonald C. | 2013 | Qualitative Research | CASP | Low to Moderate | Potential selection bias due to voluntary participation, lack of discussion on researcher reflexivity, and conference-based recruitment |
| 3 | Podcasting in medical education: a review of the literature | Cho D. | 2017 | Review of the literature | NOS | Moderate | Lack of a formal risk of bias assessment for included studies, exclusion of video-based podcasts, and unclear inter-reviewer agreement on data extraction |
| 4 | Learning through listening: a scoping review of podcast use in medical education | Kelly JM. | 2022 | Scoping review | NOS | Moderate | No risk of bias assessment included in review |
| 5 | Health care professional and caregiver attitudes toward and usage of medical podcasting: questionnaire study | Lee C. | 2022 | Survey study | NOS | Moderate | Possible recall bias in survey responses |
| 6 | Short-duration podcasts as a supplementary learning tool | Prakash S. | 2017 | Pre- and post-intervention | Cochrane RoB 2 | Moderate to High | Lack of randomization, potential self-selection bias, missing outcome data concerns, and possible selective reporting |
| 7 | Podcasts as an integral part of free open access medical education | Fernandes CAdS. | 2023 | Narrative review | CASP | High | Limited database search, lack of independent screening, no formal risk of bias assessment for included studies, potential selection bias |
| 8 | Depth of Anesthesia: A Podcast Project to Improve Perioperative Patient Care | Hao D. | 2021 | Descriptive analysis | NOS | High | Lack of structured methodology, significant author bias, no systematic analysis or independent validation |
| 9 | Education research: evaluating the use of podcasting for residents during EEG instruction | Bensalem-Owen M. | 2011 | Pre- and post-intervention | Cochrane RoB 2 | Low to Moderate | Lack of randomization, small sample size, historical control comparison, potential self-selection bias, no pre-registered protocol |
| 10 | Evaluation of a delirium awareness podcast for undergraduate nursing students in Northern Ireland: a pre−/post-test study | Mitchell G. | 2021 | Pre- and post-intervention | Cochrane RoB 2 | Moderate to High | Lack of randomization, no control group, potential self-selection bias, uncertain response rate, no correction for multiple statistical tests |
| 11 | Why not a podcast? Assessing narrative audio and written curricula in obstetrical neurology | Roth J. | 2020 | Randomized controlled trial | Cochrane RoB 2 | Moderate | No validation of assessment tools, potential self-selection bias, lack of pre-registration, no adjustment for multiple statistical tests |
| 12 | Student-Led Medical Education Podcast Improves Academic Preparedness, Increases Sense of Belonging, and Enhances Wellness | Rachel M. | 2022 | Survey | NOS | Moderate to High | Self-selection bias, non-response bias, no control for confounders, and self-reported data subject to bias |
| 13 | Characteristics of drug-related podcasts and this medium’s potential as a pharmacy education tool | Kane SP. | 2019 | Descriptive analysis | NOS | Moderate | Exclusion of video-based and non-English podcasts, lack of control for confounders, subjective interpretation of podcast content. |
| 14 | How to create and evaluate a resident-led audio program: six clinical podcasts for medicine house staff | Ghiathi C. | 2020 | Pre- and Post-Intervention Study | Cochrane RoB 2 | Moderate to High | Lack of randomization, no control group, potential selection bias, reliance on self-reported measures, unvalidated survey tools |
| 15 | Listen up: a systematic review of the utilization and efficacy of podcasts for medical education | Caldwell KE. | 2024 | Systematic review | NOS | Moderate | no publication bias analysis, unclear whether data extraction was independently verified |
| 16 | Live lecture versus video podcast in undergraduate medical education: A randomised controlled trial | Schreiber BE. | 2010 | Crossover randomized controlled trial | Cochrane RoB 2 | Low to Moderate | study design is strong, but lack of transparency regarding missing data and selective reporting concerns introduce minor biases |
| 17 | Creation of a Student-Run Medical Education Podcast: Tutorial | Kevin John M. | 2021 | Descriptive study | CASP | High | No formal research methodology, significant author bias, lack of empirical evaluation, no independent assessment |
| 18 | Effect of Interpolated Questions on Podcast Knowledge Acquisition and Retention | Michael W. | 2020 | Double-blind randomized controlled trial | Cochrane RoB 2 | Low | Potential concerns regarding missing data handling, but overall study integrity remains strong |
| 19 | Educational Impact of a Podcast Covering Vitreoretinal Topics | Michael J. | 2019 | Cross-sectional survey | NOS | Moderate to High | Self-selection bias, non-response bias, lack of confounder control, self-reported measures |
| 20 | Texting brief podcasts to deliver faculty development to community-based preceptors | Bernstein J. | 2018 | Pre-post-survey | Cochrane RoB 2 | Moderate to High | Lack of randomization, self-selection bias, subjective self-reported measures, potential attrition bias |
| 21 | A new podcast for reducing stigma against people living with complex mental health issues | Alayed YN. | 2023 | Mixed methods study (Cross-Sectional Survey & Qualitative Focus Groups) | NOS, CASP | NOS: Moderate to High  CASP: Low to Moderate | The survey component is more prone to bias due to self-selection and non-response issues.  The focus groups provide more reliable qualitative insights but are still influenced by researcher bias and limited applicability |
