# Supplementary material for: Exploring the impact, challenges, and integration of podcasts in patient education: a systematic review
Source: BMC Med Educ. 2025 May 12;25:690. doi: 10.1186/s12909-025-07217-4 (PMC12067963; doi:10.1186/s12909-025-07217-4)
Supplement: Supplementary file 3 — Supplementary Material 3 [file 12909_2025_7217_MOESM3_ESM.docx]

**Supplementary File**

***Table 2 - Table of characteristics of included studies***

| **No.** | **Title** | **First Author** | **Publication Year** | **Journal** | **Study Design** | **Outcome** |
| --- | --- | --- | --- | --- | --- | --- |
| 1 | An Evaluation of Emergency Medicine Core Content Covered by Podcasts | Riddell J. | 2023 | Western Journal of Emergency Medicine | Retrospective review | Imbalanced coverage of EM core topics; gaps in musculoskeletal, hematology, and environmental content. |
| 2 | Brain development, mental health and addiction: a podcast series for undergraduate medical education | J. MacDonald C. | 2013 | Interactive Technology and Smart Education | Qualitative Research | Positive reception for podcast use in medical education; suggestions for improvement implemented to meet user needs. |
| 3 | Podcasting in medical education: a review of the literature. Korean journal of medical education | Cho D. | 2017 | Korean journal of medical education | Review of the literature | Podcasts are feasible and accepted by learners; limited evidence on efficacy and best practices; need for rigorous studies on behavior and patient outcomes. |
| 4 | Learning through listening: a scoping review of podcast use in medical education | Kelly JM. | 2022 | Academic Medicine | Scoping review | Podcasts valued for portability, efficiency; improve knowledge and behavior; no data on patient outcomes. |
| 5 | Health care professional and caregiver attitudes toward and usage of medical podcasting: questionnaire study | Lee C. | 2022 | JMIR pediatrics and parenting | Survey study | Health professionals engage more than parents; both value accuracy, transparency, and credibility. |
| 6 | Short-duration podcasts as a supplementary learning tool: perceptions of medical students and impact on assessment performance | Prakash S. | 2017 | BMC medical education | Pre- and post-intervention | Short podcasts well-received; useful for revision; no overall score difference. |
| 7 | Podcasts as an integral part of free open access medical education | Fernandes CAdS. | 2023 | Revista Brasileira de Educação Médica | Narrative review | Podcasts are a promising complementary tool; need evidence-based guidelines for development. |
| 8 | Depth of Anesthesia: A Podcast Project to Improve Perioperative Patient Care | Hao D. | 2021 | Transl Perioper & Pain Med | Descriptive analysis | Podcasts promote evidence-based practice, critical thinking, and global engagement in anesthesia. |
| 9 | Education research: evaluating the use of podcasting for residents during EEG instruction: a pilot study. | Bensalem-Owen M. | 2011 | Neurology | Pre- and post-intervention | Podcast training as effective as traditional lectures for EEG knowledge improvement. |
| 10 | Evaluation of a delirium awareness podcast for undergraduate nursing students in Northern Ireland: a pre−/post-test study | Mitchell G. | 2021 | BMC nursing | Pre- and post-intervention study with questionnaires assessing knowledge and confidence. | Podcast improved nursing students' delirium knowledge and confidence significantly. |
| 11 | Why not a podcast? Assessing narrative audio and written curricula in obstetrical neurology | Roth J. | 2020 | Journal of Graduate Medical Education | Randomized controlled trial | Podcasts and written materials improved knowledge equally; podcasts rated more enjoyable. |
| 12 | Student-Led Medical Education Podcast Improves Academic Preparedness, Increases Sense of Belonging, and Enhances Wellness | Rachel M. | 2022 | Research Square | Survey | Student-run podcasts reduce stress, increase preparedness, and foster belonging. |
| 13 | Characteristics of drug-related podcasts and this medium’s potential as a pharmacy education tool | Kane SP. | 2019 | American Journal of Pharmaceutical Education | Descriptive analysis | Drug-related podcasts are accessible but lack quality control; pharmacists underrepresented. |
| 14 | How to create and evaluate a resident-led audio program: six clinical podcasts for medicine house staff | Ghiathi C. | 2020 | MedEdPORTAL | Pre- and Post-Intervention Study | Podcasting can be a resource for resident education and an opportunity for residents to grow as medical educators. |
| 15 | Listen up: a systematic review of the utilization and efficacy of podcasts for medical education | Caldwell KE. | 2024 | Global Surgical Education-Journal of the Association for Surgical Education | Systematic review | Podcasts widely used but efficacy unclear; limited high-quality evidence supports their use. |
| 16 | Live lecture versus video podcast in undergraduate medical education: A randomised controlled trial | Schreiber BE. | 2010 | BMC medical education | Crossover randomized controlled trial | No difference in knowledge recall; students preferred live lectures over podcasts. |
| 17 | Creation of a Student-Run Medical Education Podcast: Tutorial | Kevin John M. | 2021 | JMIR medical education | Descriptive study | Student-run podcasts foster professional identity and near-peer mentoring; widely accessible. |
| 18 | Effect of Interpolated Questions on Podcast Knowledge Acquisition and Retention: A Double-Blind, Multicenter, Randomized Controlled Trial | Michael W. | 2020 | Annals of Emergency Medicine | Double-blind randomized controlled trial | Interpolated questions in podcasts improve knowledge retention, especially for highlighted material. |
| 19 | Educational Impact of a Podcast Covering Vitreoretinal Topics: 1-Year Survey Results | Michael J. | 2019 | Journal of VitreoRetinal Diseases | Cross-sectional survey | Podcasts valued for staying updated and learning; no replacement for traditional methods. |
| 20 | Texting brief podcasts to deliver faculty development to community-based preceptors in longitudinal integrated clerkships | Bernstein J. | 2018 | MedEdPORTAL | Pre- and post-survey | Podcasts improved teaching practices; well-received by community-based preceptors. |
| 21 | A new podcast for reducing stigma against people living with complex mental health issues: Co-design study | Alayed YN. | 2023 | Journal of Survey in Fisheries Sciences | Mixed methods study (Cross-Sectional Survey & Qualitative Focus Groups) | reduce stigma through lived experience narratives. |
